# The Galaxy platform for accessible, reproducible, and collaborative data analyses: 2024 update

**DOI:** 10.1093/nar/gkae410

**Published:** 2024-05-20

**Authors:** The Galaxy Community, Linelle Ann L Abueg, Enis Afgan, Olivier Allart, Ahmed H Awan, Wendi A Bacon, Dannon Baker, Madeline Bassetti, Bérénice Batut, Matthias Bernt, Daniel Blankenberg, Aureliano Bombarely, Anthony Bretaudeau, Catherine J Bromhead, Melissa L Burke, Patrick K Capon, Martin Čech, María Chavero-Díez, John M Chilton, Tyler J Collins, Frederik Coppens, Nate Coraor, Gianmauro Cuccuru, Fabio Cumbo, John Davis, Paul F De Geest, Willem de Koning, Martin Demko, Assunta DeSanto, José Manuel Domínguez Begines, Maria A Doyle, Bert Droesbeke, Anika Erxleben-Eggenhofer, Melanie C Föll, Giulio Formenti, Anne Fouilloux, Rendani Gangazhe, Tanguy Genthon, Jeremy Goecks, Alejandra N Gonzalez Beltran, Nuwan A Goonasekera, Nadia Goué, Timothy J Griffin, Björn A Grüning, Aysam Guerler, Sveinung Gundersen, Ove Johan Ragnar Gustafsson, Christina Hall, Thomas W Harrop, Helge Hecht, Alireza Heidari, Tillman Heisner, Florian Heyl, Saskia Hiltemann, Hans-Rudolf Hotz, Cameron J Hyde, Pratik D Jagtap, Julia Jakiela, James E Johnson, Jayadev Joshi, Marie Jossé, Khaled Jum’ah, Matúš Kalaš, Katarzyna Kamieniecka, Tunc Kayikcioglu, Markus Konkol, Leonid Kostrykin, Natalie Kucher, Anup Kumar, Mira Kuntz, Delphine Lariviere, Ross Lazarus, Yvan Le Bras, Gildas Le Corguillé, Justin Lee, Simone Leo, Leandro Liborio, Romane Libouban, David López Tabernero, Lucille Lopez-Delisle, Laila S Los, Alexandru Mahmoud, Igor Makunin, Pierre Marin, Subina Mehta, Winnie Mok, Pablo A Moreno, François Morier-Genoud, Stephen Mosher, Teresa Müller, Engy Nasr, Anton Nekrutenko, Tiffanie M Nelson, Asime J Oba, Alexander Ostrovsky, Polina V Polunina, Krzysztof Poterlowicz, Elliott J Price, Gareth R Price, Helena Rasche, Bryan Raubenolt, Coline Royaux, Luke Sargent, Michelle T Savage, Volodymyr Savchenko, Denys Savchenko, Michael C Schatz, Pauline Seguineau, Beatriz Serrano-Solano, Nicola Soranzo, Sanjay Kumar Srikakulam, Keith Suderman, Anna E Syme, Marco Antonio Tangaro, Jonathan A Tedds, Mehmet Tekman, Wai Cheng (Mike) Thang, Anil S Thanki, Michael Uhl, Marius van den Beek, Deepti Varshney, Jenn Vessio, Pavankumar Videm, Greg Von Kuster, Gregory R Watson, Natalie Whitaker-Allen, Uwe Winter, Martin Wolstencroft, Federico Zambelli, Paul Zierep, Rand Zoabi

## Abstract

Galaxy (https://galaxyproject.org) is deployed globally, predominantly through free-to-use services, supporting user-driven research that broadens in scope each year. Users are attracted to public Galaxy services by platform stability, tool and reference dataset diversity, training, support and integration, which enables complex, reproducible, shareable data analysis. Applying the principles of user experience design (UXD), has driven improvements in accessibility, tool discoverability through Galaxy Labs/subdomains, and a redesigned Galaxy ToolShed. Galaxy tool capabilities are progressing in two strategic directions: integrating general purpose graphical processing units (GPGPU) access for cutting-edge methods, and licensed tool support. Engagement with global research consortia is being increased by developing more workflows in Galaxy and by resourcing the public Galaxy services to run them. The Galaxy Training Network (GTN) portfolio has grown in both size, and accessibility, through learning paths and direct integration with Galaxy tools that feature in training courses. Code development continues in line with the Galaxy Project roadmap, with improvements to job scheduling and the user interface. Environmental impact assessment is also helping engage users and developers, reminding them of their role in sustainability, by displaying estimated CO_2_ emissions generated by each Galaxy job.

## Graphical Abstract

**Graphical Abstract:**
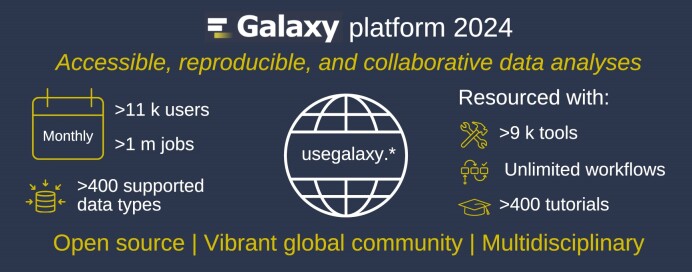

## Introduction

User demand for an easily accessible data analytics service, deployed on computing infrastructure capable of meeting the needs of complex computing in research, has resulted in the Galaxy Project supporting, in its 19th year of ongoing operation, a rapid increase in throughput globally (1,2). Galaxy provides analytical tools that can be used individually or linked into complex workflows with intermediate data outputs capable of triggering logic conditionals within the workflow. Recent enhancements allow researchers to run workflows on data of variable quality, and have the workflow buffered to systematically explore experimental variability (https://gxy.io/GTN:T00164). Large scale research is necessarily collaborative, and Galaxy's capacity to both securely share and publish data and workflows supports efficient collaboration, training, and data reuse. Recent changes to the Galaxy user interface discussed below have made sharing more visible.

Collectively, the usegalaxy.* services in the United States, Australia, and Europe have amassed >500 000 registered users, and supports >11 000 individual users running >1 000 000 jobs on average each month of 2023. Usegalaxy.* service statistics are publicly available at https://status.galaxyproject.org/, with detailed operational data for Australia and Europe at https://stats.usegalaxy.org.au/ and https://stats.galaxyproject.eu/, respectively. Users have access to >9000 scientific tools, supporting >400 different types of input data, enabling a wide variety of analyses in both the life and physical sciences, including astronomy, genomics, proteomics, metabolomics, materials science, imaging, and cytometry. Efficient, reproducible complex analytical pipelines can be created by joining tools from any domain with ‘noodles’ on the workflow canvas. Analysis outputs can be explored with >50 types of inbuilt visualizations, and a simple URL can be shared with collaborators, which encapsulates all the data, analyses settings, tool versions, and workflows needed for replication.

## Research driven Solutions

Galaxy makes thousands of third-party open-source analysis packages easy to use, and interoperable without any user supplied code. For any new analysis package to become a tool, a developer prepares a Galaxy wrapper once, and uploads it to the sharable Galaxy global tool ‘appstore’ called the Galaxy Toolshed (https://toolshed.g2.bx.psu.edu/). Each Galaxy service supports a core common tool set, and offers a wide range of other tools, the exact combination of which is driven by user demand. The tools are then categorized by scientific use and/or datatypes involved. Further, the option to host tools on Galaxy is conditional on the tools’ computational needs (e.g. GPGPUs, high-memory), licence stipulations of tool use, and the ability for it to be wrapped as a standard or interactive tool, all of which have been improved in the latest Galaxy updates (https://docs.galaxyproject.org/en/master/releases/index.html).

### Galaxy Labs/sites/subdomains

The Galaxy Toolshed now hosts over 9500 distinct and modular software packages, i.e. tools, available to Galaxy Administrators for easy installation on any Galaxy service. The breadth of analytical options available as installed tools can add value for end users but can also be overwhelming. User feedback, both active formal UX documentation and passive user-initiated feedback, have identified that even a fraction of the tools hosted on a Galaxy service can confuse the process of finding any specific tool. For example, a user looking for a singular tool on the usegalaxy.* servers have to navigate through sets of 1770 (Galaxy US), 3320 (Galaxy EU) and 1730 (Galaxy AU) tools. This can be daunting, even when considering the help provided through tool categorisation and EDAM ontology labels (3).

**Figure 1.**
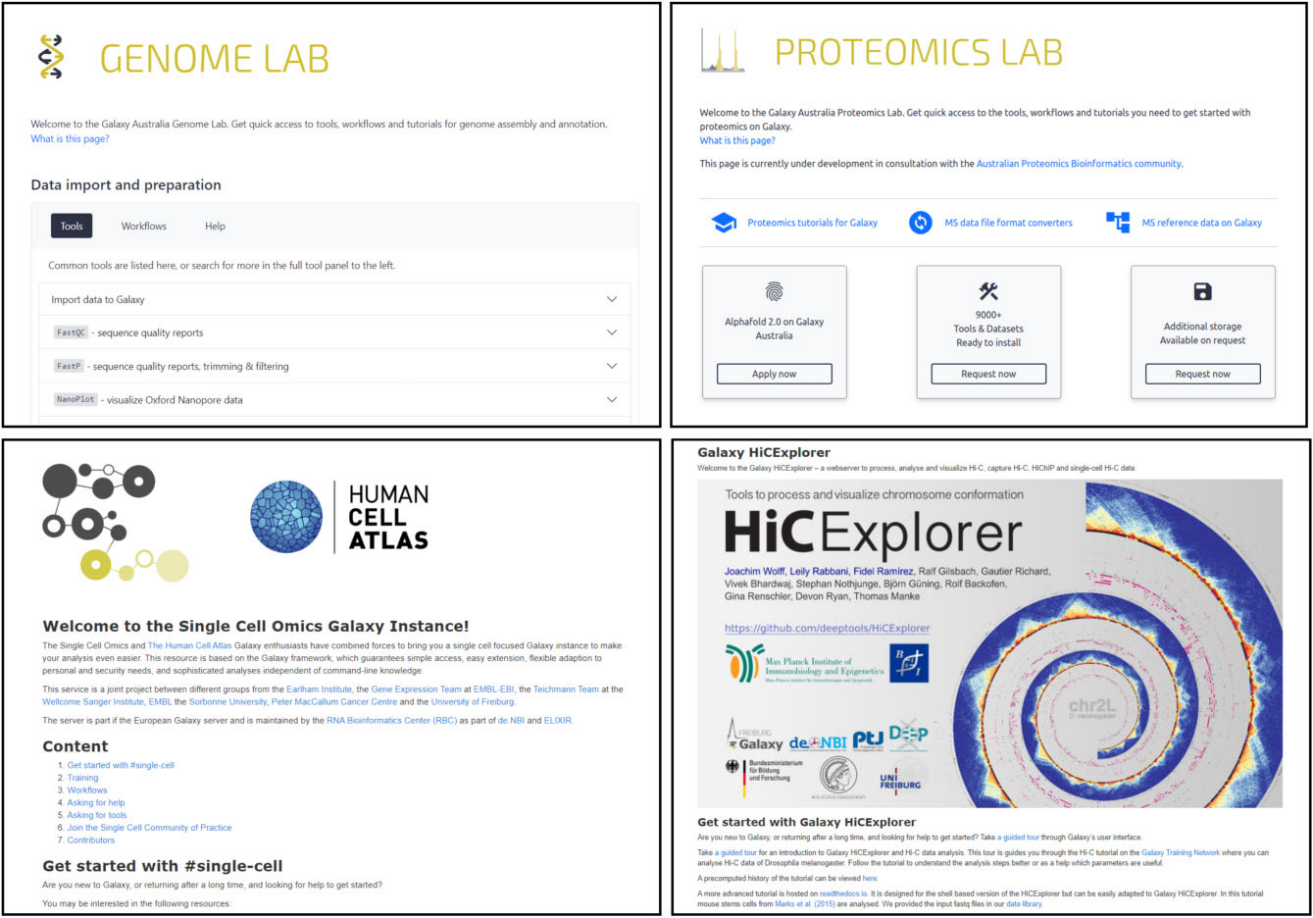
Examples of Galaxy Labs/subdomains. Researchers can quickly access a concentration of domain-specific tools, workflows, support, and training through Galaxy Labs or Galaxy subdomains. Top: the Genome Lab and Proteomics Lab on Galaxy Australia, https://genome.usegalaxy.org.au and https://proteomics.usegalaxy.org.au. Bottom: the Single Cell Omics subdomain on Galaxy Europe, https://singlecell.usegalaxy.eu/ and https://hicexplorer.usegalaxy.eu.

Galaxy Europe first provided a solution to empower researchers with a common interest or who undertake a set of activities frequently. The subdomains focus on a particular research domain or technology modality (Figure 1). The content, tools and resources available are ‘tailored’ to each domain - i.e. making sure that the resources are a good fit for routine real-world research practice. Galaxy Australia also now makes use of this option, naming its offering *Galaxy Labs* (Figure 1). These labs offer a concentration of tools, workflows, and resources allowing new and regular practitioners of that field ready access to the most common options they need, whilst still offering all the other Galaxy features they could use. Importantly, a user logged in on a Galaxy offering labs/subdomains has full access to their data (histories), workflows, shared data, across all labs and the main service page. Galaxy Labs also align with Galaxy Project's strategic initiative to support global research consortia, such as the Vertebrate Genome Project (VGP) (4) and Earth BioGenome Project (EBP) (5). Important regional examples include the adoption of Galaxy within the European Partnership for the Assessment of Risks from Chemicals (EU-PARC; https://www.eu-parc.eu/) (6), WP4 Task 4.3.1.d, as a platform of choice for processing small molecule mass spectrometry datasets, and separately for processing of mass spectrometry datasets generated via the Czech node (coordinator) of the European Environmental Exposure Assessment Research Infrastructure (EIRENE-CZ; https://www.eirene-ri.eu/). In Australia, the Threatened Species Initiative uses Galaxy Australia as its primary genomics analytical service (https://threatenedspeciesinitiative.com/genome-assembly/).

### GPGPU-supported tools

Increased tool complexity and capacity is evident in Galaxy through recent tool offerings utilising GPGPU infrastructure. Tool execution environment resourcing is not a consideration for Galaxy users but is easily configured by Galaxy infrastructure providers. GPGPU-based tools (AlphaFold2.0/multimer (7), ChatGPT (8) and AI-based tools run in JupyterLab (7)) are highly requested and are delivered through local access to GPGPU or through commercial cloud provision, across the usegalaxy.* services. In the case of Galaxy Australia, this work necessitated the deployment of a Pulsar on the Australian instance of Microsoft Azure. The demand for AlphaFold2.0/multimer in Australia has branched into utilization of NVIDIA GPUs and more recently the code has been adapted to also work on AMD GPUs, to decouple the tool use from a specific set of hardware requirements and to allow the tool to be deployed on a greater range of worker node configurations. Within the US, Galaxy leverages GPGPU and other compute resources via the NSF-sponsored ACCESS-CI network, and now supports the widely used AlphaFold/CollabFold algorithms (9), GPU-based signal processing tools for ONT sequencing data (10), and other highly requested GPU tools. In support of global genome assembly consortiums, the GPGPU tool Helixer has been deployed onto usegalaxy.* services and is expected to drive GPGPU utilisation in support of programs such as the European Reference Genome Atlas (ERGA, https://www.erga-biodiversity.eu/) and VGP. Future Galaxy Project updates are predicted to increasingly utilize GPGPU and tensor processing unit (TPU)-dependent tools.

### Licenced tools

A primary driver for Galaxy's success has been its foundation in the principles of open-source development. However, this has limited the implementation of tools for Galaxy that have a non-open-source licensing arrangement. In some cases, development of an open-source equivalent has minimal delay after commercial solutions become available; MaxQuant (11) as a pan-proteomics tool is one such example. In rare cases where open-source development cannot keep pace with commercial solutions or user demands, Galaxy has increasingly turned to offering licenced options, for example: CellRanger and FGeneSH++ (12,13). Working in the interest of users, this provides a solution, which is the primary goal. It involves a local administrative burden in controlling access and licence agreements. The Galaxy community hopes that sufficient evidence of utility will make a compelling case for vendors to reconsider their licensing agreements to increase exposure to their tools (and brand) through Galaxy usage that acknowledges their contribution.

### Discoverability

Any individual tool or workflow is only useful if it can be discovered in the first place, and this discoverability extends to the core functions of the software. In effect, a researcher should be able to discover an analytical solution using either the specific software name and associated metadata, or on the basis that they are seeking a particular functionality. In either case, the discovery of tool and workflow metadata has become increasingly important for both users and administrators of Galaxy. Existing capacity to share this metadata across life science domains that make use of Galaxy will greatly increase the scientific impact of these improvements. A solution for individual tools recently developed, with an initial focus on microbiology, is at https://galaxyproject.org/community/sig/microbial/#tools. This interactive searchable table combines metadata sourced from Galaxy tool wrappers (via Planemo), Galaxy instance APIs (adding tool availability), ELIXIR bio.tools (https://bio.tools/) (adding EMBRACE Data And Methods’ (EDAM terms (3)), BioConda (14) (checking if tools are current), and a community-curated worksheet (flagging tools to be excluded). Galaxy already supports versioning through the Workflow editor (i.e. the canvas). However, the need to share workflows collaboratively using persistent identifiers, and to align with the FAIR principles for research software (15), has led to integration with workflow registries like WorkflowHub (https://workflowhub.eu/) and Dockstore (16). From WorkflowHub, researchers can select a ‘Run on usegalaxy.eu’, which triggers workflow import to Galaxy Europe; from Dockstore, a Galaxy workflow can be imported into any of the three major usegalaxy.* servers. Within Galaxy, a user can search either WorkflowHub or Dockstore using their GA4GH Tool Registry Service (TRS) implementations (17), alongside existing options for workflow import from local files and public URLs.

## Galaxy Training Network

A long-standing and core feature of Galaxy is its ability to deliver accessible, convenient and highly reproducible training, in scheduled trainer-supported programs, or as self-directed active learning. The Galaxy Training Network (GTN; http://training.galaxyproject.org) (18) is the complementary service that hosts tutorials, guides, and infrastructure for feedback/improvements on content. The GTN offers >400 tutorials across 25 scientific and 6 technical topics, written by >325 contributors.

### GTN materials

The GTN strives to maintain tutorials, keeping them synchronized with changes in research practice, updating 366 tutorials (90%) in the past year alone. Nine new topics have been added including Single Cell Analysis, SARS-CoV-2, One Health, Evolution, Materials Science, and Synthetic Biology. The topic focus has expanded from purely scientific topics to include broader topics such as the new ‘FAIR data analysis’ topic, which aims to guide learners to make their data and workflows FAIRer via hands-on tutorials. Additionally, a ‘Data science’ topic has been added covering basics of Python, R, SQL and snakemake (19) using interactive notebooks which can be run inside Galaxy as interactive tools, or independently of Galaxy. This work has been supported by the addition of over 70 new contributors to the GTN in the past 2 years, which alongside the existing contributor community have added a total of 133 tutorials to Galaxy since April 2022. Finally, an exploratory collaboration with AI4Life (https://ai4life.eurobioimaging.eu/) has produced several slide decks introducing learners to the BioimageModelZoo. The GTN hosts these training materials on custom-branded pages to provide scientific consortia with a way to host their training materials with minimal maintenance burden, even though they have no direct connection with Galaxy.

### GTN framework

Alongside the steady increase in tutorial numbers, the framework itself is also continually improved. New features are developed, to support the utility of the GTN for both learners and educators. These include:

- ***Automated video slides***: When comprehensive speaker notes are provided with a slide deck, the GTN framework will automatically create a video lecture based on the slides, using automated text-to-speech (TTS) software. This is a useful resource for learners, as well as educators preparing to teach the slide deck. Finally, the maintenance burden is significantly lower than for live videos, as any change in the slides or speaker notes will trigger an automatic rebuild of the videos.
- ***Defining learning pathways***: Learning paths describe a journey around a topic or set of topics, that guide learners from introductory materials to increasingly advanced tutorials. These learning pathways can include materials from different GTN topics and allow grouping into modules. Learning pathways support learners trying to find suitable tutorials to achieve their learning objectives, as well as educators in crafting a curriculum. These learning pathways have been typically based on week-long training courses organised by the community.
- ***Support for modular lessons/choose-your-own-adventure tutorials***: It is now possible to present learners with a choice and depending on their choice, the tutorial contents are changed. This option has been used in various ways, for example to offer a choice of different alignment tools in the RNA-seq tutorial, or to adjust the level or length of the 16S metagenomics tutorial, where users can choose if they want an higher-level view of the topic by running a set of five workflows, or if they want to dive into the complexity of analytical options and file formats, and run each of the 30+ steps manually.
- ***Support for interactive notebook-based coding tutorials***: These tutorials can be viewed either in the traditional GTN view as a static web page, where learners launch Rstudio or Jupyter and perform the hands-on instructions. In addition, the GTN framework can also convert these tutorials into fully-fledged Jupyter notebooks, where the user can perform the hands-on tasks directly inside the tutorial notebook, while also having the full tutorial (scientific explanations, question boxes, etc.) loaded in the notebook.
- ***GTN support inside Galaxy***: Accessing GTN materials is now possible directly from within the Galaxy Web interface. When the materials are accessed in this manner, it enables the GTN’s click-to-run workflows and tools integration, whereby users can click on tool/workflow names in the tutorials to automatically open them inside their open Galaxy session.
- ***Pan GTN improvements***: Persistent identifiers (PURLs) for tutorials, GTN API, support for manually curated tutorial translations, website themes and improved search functionality.

### GTN events

In addition to the frequent training events organized by the broader Galaxy community, the large-scale global Galaxy Smörgåsbord training event, started in 2021, has been repeated in the past two years, attracting 3082 (2022) and 2965 (2023) registrations. These events offered fully remote, highly flexible, asynchronous, video-based learning with support from the Galaxy community on Slack. Learners could design their own program based on their own experience and interests, and determine their own schedules based on their own time constraints.

### Training infrastructure as a service (TIaaS)

In support of GTN and Galaxy-based training events in terms of compute resources, we have developed and released Training-Infrastructure-as-a-Server (TIaaS) (20). TIaaS allows Galaxy administrators to reserve compute resources for training events, to minimise queue times for participants. Educators additionally get access to a dashboard that shows an overview of the status of participant's tool runs, allowing them to easily view progress and identify problems, even in a remote teaching setting. In the past 70 months, over 500 training events with over 24 000 learners have used TIaaS for Galaxy training.

## User-focused features and enhancements

The increasingly complex offerings within Galaxy have been matched by the improvements to the usability of Galaxy, through the application of user-driven design (UXD). Beginning in the GTN and in recognition of Galaxy's aim to cater to all researchers from all regions of the global and with all manner of accessibility needs, Galaxy has deployed several features to enhance accessibility. These include:

- ***Legibility***: Both the GTN and Galaxy have adopted Atkinson Hyperligible, a font designed by the Braille Institute (https://brailleinstitute.org/freefont) that aims to improve legibility for low-vision readers by making letterforms easy to recognize even when blurry. This change helps us meet our goal of an accessible platform and making data science accessible for everyone, including those with visual impairments. The GTN has a longstanding commitment to accessibility and regularly tests its interface with a screen reader which has helped catch numerous accessibility issues that would not be noticed by sighted learners, all to the benefit of a larger more inclusive community. Galaxy has recently launched a similar effort to improve the screen reader accessibility of its interface, making large strides in reducing the number of mouse-only workflows and improving keyboard navigation.
- ***Colour* *schemes***: Galaxy has implemented a framework allowing for customization of system colours and the user interface, allowing individual Galaxy deployments to customise their colours to match their branding, supporting customizing logos and the masthead colouring (https://galaxyproject.org/news/2023-04-25-themes-in-galaxy). The GTN found issues in its existing implementation and separated out cosmetic changes from the more important accessibility axes of automatic dark/light mode responding to the user's browser preferences for colour scheme and contrast, allowing users to choose any cosmetic theme separately from their visual needs.
- ***Pan-Galactic Tool Search***: The GTN has begun collecting lists of publicly shared workflows (https://training.galaxyproject.org/training-material/workflows/list.html) and tools (https://gxy.io/GTN:N00055) across public Galaxy services, enabling both learners and researchers to more easily discover both what resources and where those resources are available to access.
- *‘* ***Click to run*** *’*: WorkflowHub.eu and Dockstore are both integrated into Galaxy via the GA4GH TRS API which gives users a ‘click to run’ experience. They can identify a workflow in their preferred hub, and then with a click (or two) be redirected to their preferred Galaxy to launch the workflow. Within the GTN we implemented a similar feature, any workflows inside the GTN are likewise launchable directly in the user's preferred Galaxy via the TRS API. When these links are accessed from within a Galaxy instance via the ‘Tutorial Mode’, the workflow is launched directly in the user's active Galaxy with one click. These sorts of enhancement significantly improve the learner's experience by removing barriers and distractions from following hands-on learning materials, allowing them to focus on the content and the science.
- ***Display language***: Language configuration is possible through the Localization option in Manage Preferences. Users can easily navigate Galaxy options in their preferred language, currently selecting from: Chinese, English, French, Japanese and Spanish.

## Software features and enhancements

Galaxy updates range from user interface changes to fundamental code base refreshes and best practice adoption. Herein are described the features deployed to enable all the improvements in the utility of Galaxy described above.

### Service optimization

- ***Total Perspective Vortex (TPV)***: TPV is a library for right-sizing and meta-scheduling Galaxy jobs in heterogeneous compute environments (https://doi.org/10.48550/arXiv.2312.02060). TPV allows fine-grained control over resource allocation for individual jobs, including the ability to make decisions using live resource data. A key advantage of TPV is a first-ever community-curated database of default resource requirements for nearly 1000 popular bioinformatics tools (https://github.com/galaxyproject/tpv-shared-database). This publicly available resource has recommended resource allocations and scaling rules for tools in a simple YAML format, that takes away the need for administrators to individually configure, and often guess, job resource requirements per Galaxy deployment. TPV can be easily configured on any modern Galaxy instance and has been deployed on Galaxy AU and EU, processing over ten million jobs since its initial deployment in November 2021.
- ***GA4GH support***: By supporting the APIs developed by GA4GH (17), the Galaxy Project helps to ensure that data are easily accessible and interoperable, and can be quickly and easily analyzed by researchers and clinicians. This is particularly notable in the genomics community to advance medical research and improve patient care. The collaboration between GA4GH and the Galaxy Project therefore helps to achieve the mutual goal of making genomic data a valuable resource for the benefit of humanity. Galaxy currently supports several major APIs, including the Data Repository Service (DRS) for import and export of data hosted within Galaxy; the Task Execution Service (TES) which exposes Pulsar resources to efficiently execute large scale analyses; and the Tool Registry Service (TRS) to share and distribute workflows. Galaxy also has preliminary support for Beacon, which allows for the discovery of genome data by querying if a specific variant is present in a dataset, and several other GA4GH APIs.
- ***Deferred remote dataset resolution***: Deferred datasets is a feature that allows datasets to be fetched only when the job using them is run, potentially reducing waiting times for analysis. Tools and workflows can be executed efficiently, Galaxy will download the remote dataset only when it's needed for a specific job. Since the data isn’t stored by Galaxy until required, the dataset does not contribute to a user's storage quota.

### User experience improvements

**Figure 2.**
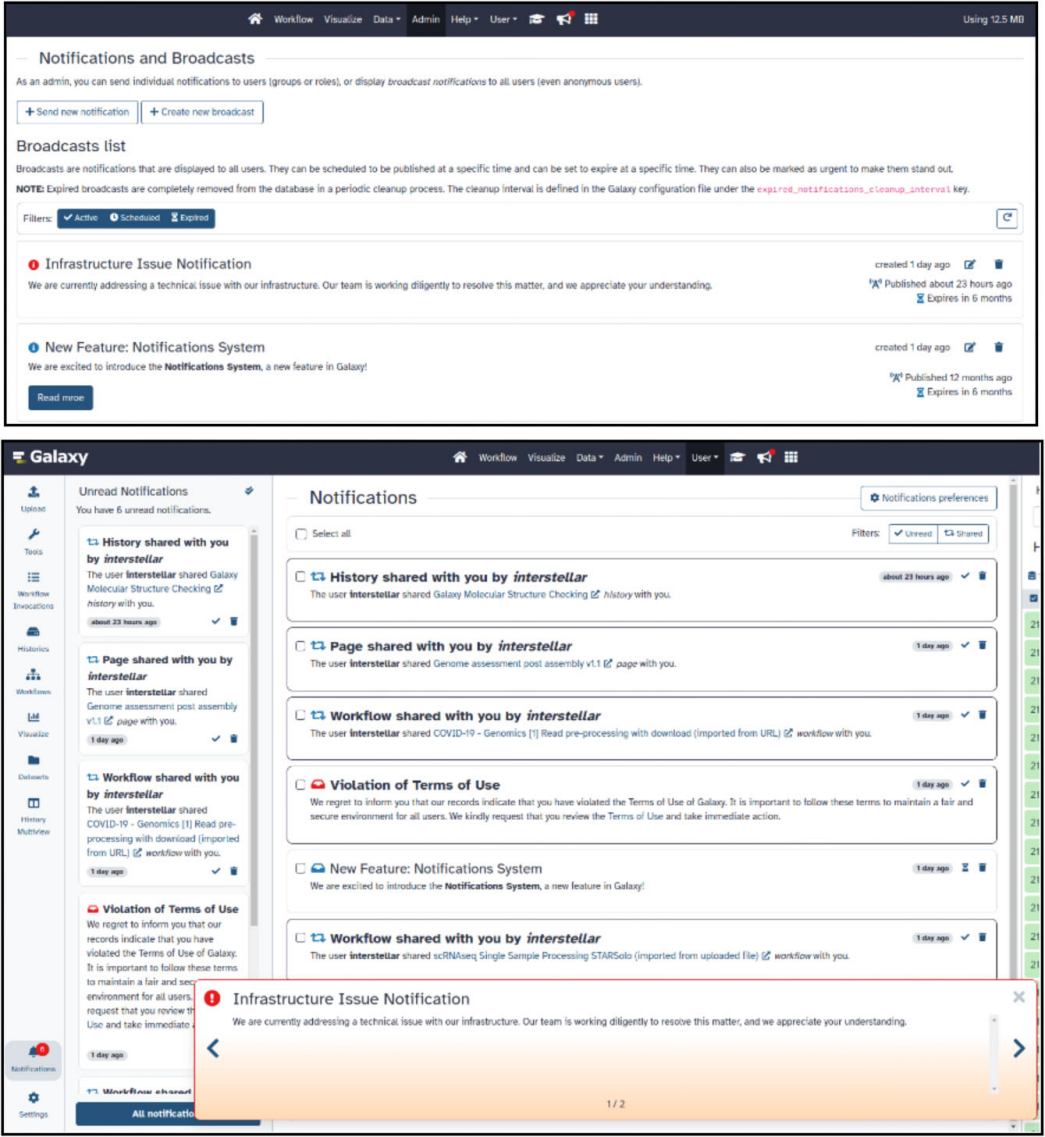
Notifications and Broadcasts Galaxy administrator and user views. Top: Galaxy administrator view for individual user Notifications and service-wide Broadcasts. Bottom: an example of the combination of Notifications and Broadcasts a user will experience.

A suite of new features have been added to Galaxy in direct support of making the Galaxy UI more intuitive and more relevant in the information displayed. These include:

- ***Notification system***: The new notification system facilitates sending notifications about a wide variety of scenarios like job completion, artifact sharing, service updates, and more. Notifications appear within the Galaxy service as red enumerated dots that navigate to the notification panel (Figure 2). Users have control over their notifications, including the option to subscribe/unsubscribe from certain types of notifications. The new notification system also supports broadcast notifications, allowing administrators to send server-wide announcements, such as server maintenance or downtime notifications.
- ***Login through* *OpenID* *Connect (OIDC) enhancements***: Galaxy tools and jobs are now able to use linked OIDC identities to carry out actions on behalf of users. This enables tools and workflows to have single-sign-on capabilities for a seamless user experience. Tool authors are able to utilise these capabilities to reduce friction for users where previously, repeated prompting for user credentials may have been required.
- ***History interface***: A core Galaxy element, the user History of input data and results, was updated to allow easier dataset input searching, quick History switching, multi-history viewer, multi-directional drag and drop in the multi-history viewer, and bulk operations such as item tagging and database key changes.
- ***Tool search***: Updated to include Advanced Tool search, allowing filtering by Section, ID and Help Text in addition to Name.

## Research data management

Galaxy stores both user data and reference data on behalf of researchers. Providing users with a clear understanding of their storage profile on a Galaxy service is important in managing the service obligations for storing data. To provide more informed data management these new features have been made available to service operators and to users to empower decision making on data storage.

**Figure 3.**
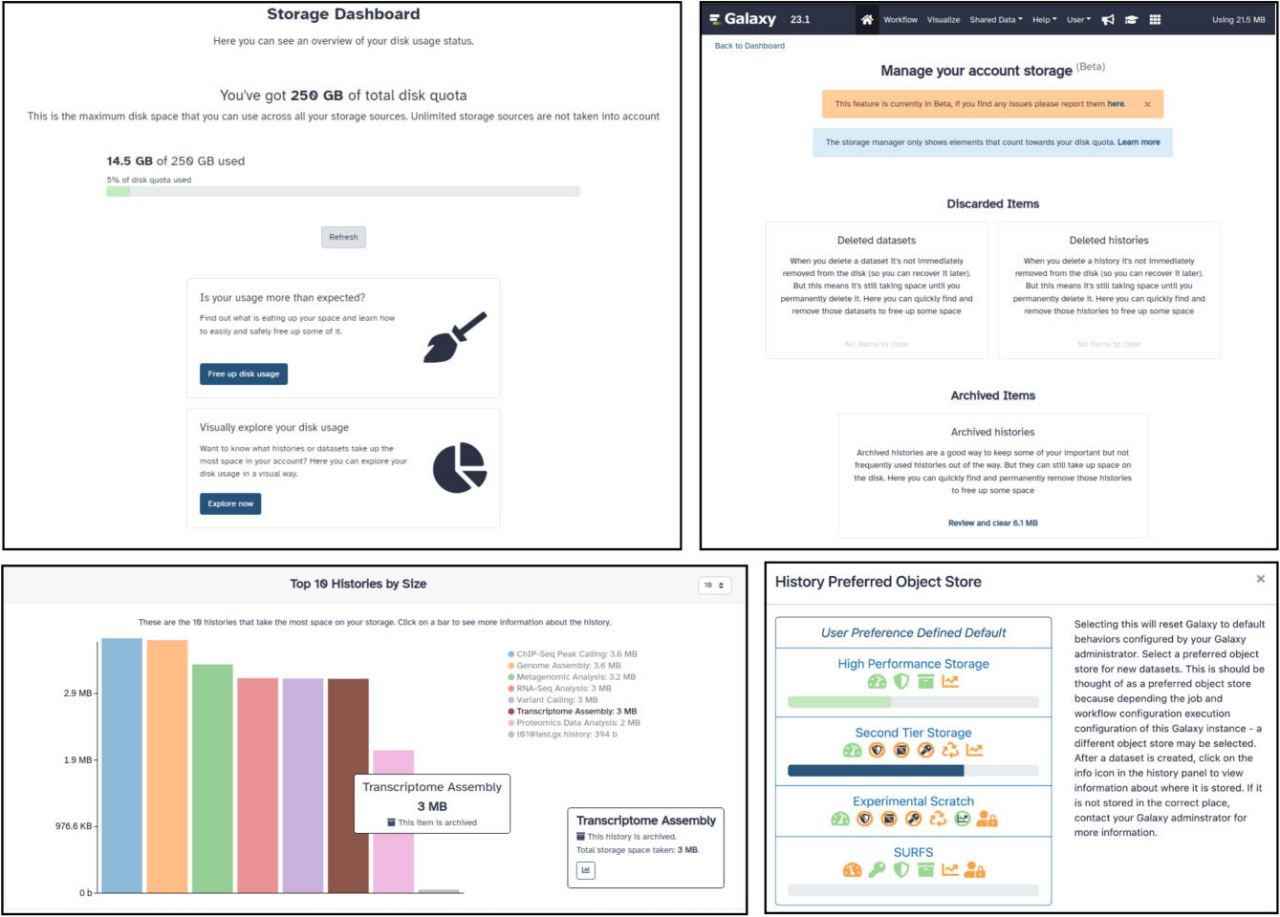
User Storage Dashboard. The User Storage Dashboard, available from release 22.05, and improved and extended on release 23.1. Top left: dashboard main overview. Top right: storage management section where a user can quickly discover and free up disk space. Bottom left: visual representation of the top 10 histories by size. Bottom right: example of possible available options to store your history objects.

**Figure 4.**
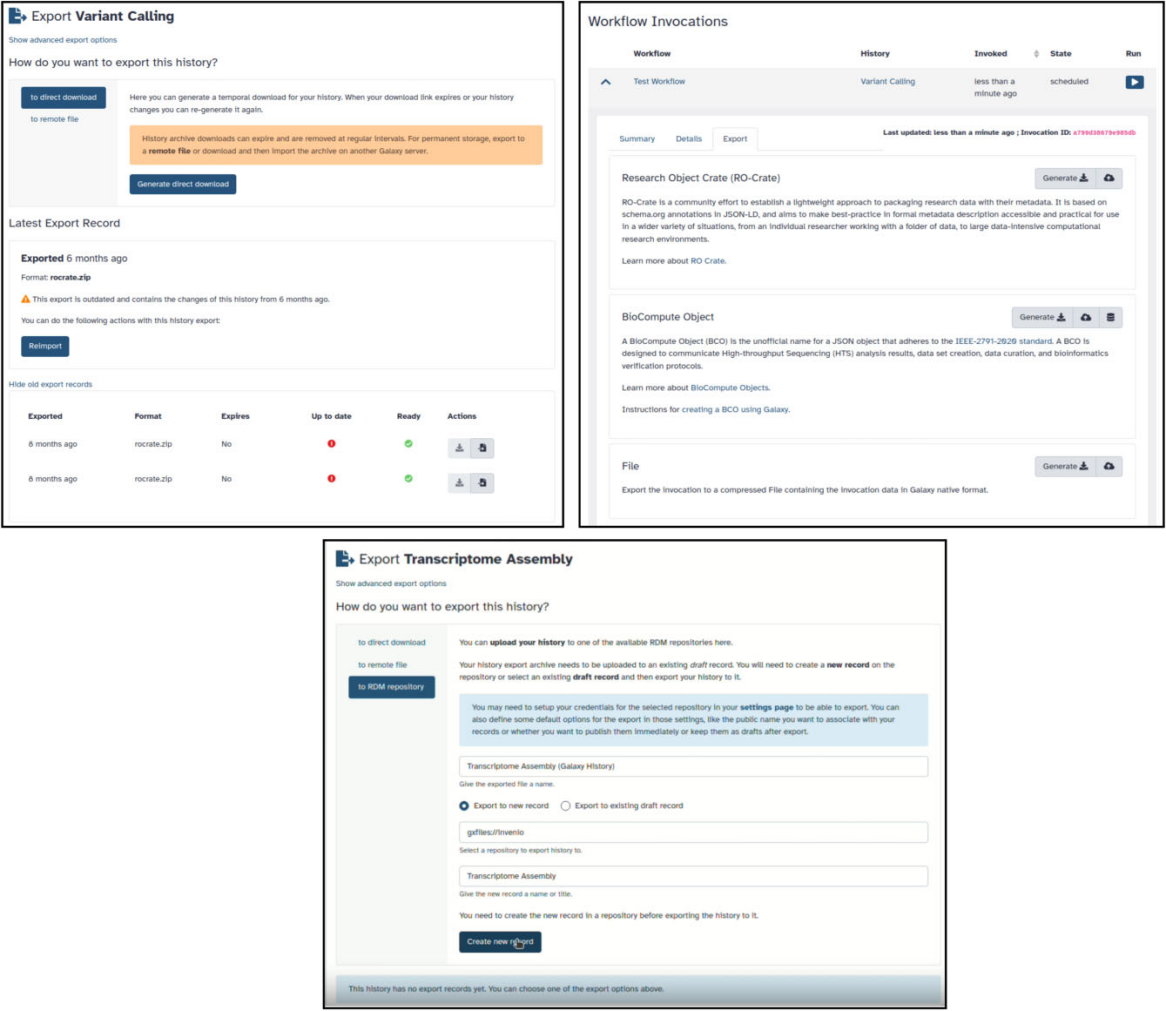
Structured data export of Galaxy objects. Top left: example of history—variant calling export to ROcrate, with tracking on prior export events. Top right: export of workflow invocation to multiple archival formats. Bottom: InvenioRDM export, supporting easy connection to Zenodo.

- ***Unused history management***: The responsibility of managing user data should reside with the user, however active reminders of approaching quota limits are usually needed to help users in this task. An option, currently deployed on Galaxy Australia, is an email alert of histories that have not been modified in the past 52 weeks and the pending deletion of such histories in 2 additional week's time. The email lists all histories with hyperlinks directly to the histories, allowing the user to easily take any action required, supported by each history size and links to support guides for downloading and preserving the history outside of Galaxy.
- ***Storage visibility***: A researcher data journey on Galaxy can include training data, optimization data, raw data and individual reference data. Each of these data classifications can be grouped by their requirement for storage/retrieval. For example, GTN training data stored on Zenodo (https://zenodo.org/communities/galaxy-training/) can be repeatedly accessed if stored temporarily on an individual Galaxy service. However raw data may need to be stored until associated results are published and data stored as required by publication. The User Storage Dashboard and Storage options help researchers manage their total storage profile. The Storage Dashboard is a central place where the user is presented with an overview of their disk storage usage (Figure 3). It also provides an easy and quick way to recover space from likely unused histories or datasets. Users can visualize the disk usage of their collected histories, with the top 10, 20 or 50 histories measured by total storage required displayed on a box plot (Figure 3). More detail can be found for individual datasets, allowing users to manage their total storage profile and what data requires export or deletion. The History Preferred Object Store storage options let the user select where to store the data depending on their needs, and differs on each public Galaxy server.
- ***Data export***: Galaxy users have been able to export History item(s) or complete histories, for the purpose of archiving or later reuse. The provenance of exports however was not tracked, and this has been improved. Histories now track when and where they were exported. Exports can be permanent or temporary. Permanent exports support quick and easy re-import into Galaxy from the ‘*File Source Plugin’* configured on the Galaxy server, such as S3, Zenodo, Dropbox. Temporary exports are short lived links that allow users to download histories, making it possible to manually upload and import them later as needed. Histories can be exported as compressed archives, or as RO-Crate objects (21), a FAIR archiving format of Research Objects based on schema.org and Bioschemas (Figure 4). Workflow invocations (or runs) can be exported to multiple formats, including RO-Crate, as well as BioComputeObjects, a standard (IEEE 2791–2020) for tracking provenance information of bioinformatics pipelines for high-throughput sequencing containing additional metadata pertaining to the workflow execution (Figure 4). Workflow export infrastructure has been extended to support new format standards, through the easy addition of new plugins to the feature. A significant example is the new InvenioRDM plugin. This plugin allows users to export/import single datasets or histories to any InvenioRDM instance (Figure 4). InvenioRDM is a turn-key research data management (RDM) repository solution developed by CERN. It is the underlying platform used by Zenodo, which in turn allows for easy import/export data from Galaxy to Zenodo.

## A vibrant global community

The Galaxy Project is supported by hundreds of active contributors to the code, tools, workflows, and training. This engagement allows the user community to play an important formal role in planning the future directions of Galaxy Project. Evidence of this engagement includes acknowledgment of Galaxy in public documentation, with >3645 papers citing Galaxy between 2022 and 2024. Followers on microblogging services are another metric. Galaxy Project reached over 14 000 followers on X (formerly Twitter), before changed rules and policies at X ceased to align with the values of participants, leading to Galaxy Project abandoning reliance on X for communication. From 2024, a multi-service approach has been adopted, with Mastodon (https://mstdn.science/@galaxyproject), Matrix (https://app.element.io/#/room/#galaxyproject_Lobby:gitter.im), BlueSky (https://bsky.app/profile/galaxyproject.bsky.social) and LinkedIn (https://www.linkedin.com/company/galaxy-project/) all destinations for Galaxy Projects messaging. Engagement also comes from clear and open communication within Galaxy Project. The governance, working group and special interest groups (SIGs) have been revised to provide appropriate custodianship of Galaxy into the future.

### Galaxy Project governance

The Galaxy Project is managed by participatory self-governance. Formal governance structures include the Galaxy Executive Board (GEB), Galaxy Community Board (GCB), Galaxy Technical Board (GTB) and the Project Management Office (PMO) (https://galaxyproject.org/community/governance/). The GEB aims to enhance its international representation by welcoming new and experienced principal investigators from diverse backgrounds. This expansion is viewed as crucial for fostering a broader and more inclusive research community with the growth of Galaxy from local projects to a global initiative.

SIGs are groups of specialists, collaborating, engaging with and contributing to Galaxy, through development and sharing of specialized resources, and by clarifying and contributing their longer term needs to Project planning (https://galaxyproject.org/community/sig/). Entirely driven by user communities, SIGs have recently been reorganized extensively to accommodate growth, emerging from community groups, self-identified by language or geographic regions (e.g. GTÑ-Español), scientific projects (e.g. responses to COVID-19), and communities of shared research practice, such as single-cell analyses methods.

These SIGs have self-organized into the GCB, to establish best practices, develop infrastructure and guidelines for themselves, and build user representation. The goal is to streamline community efforts and provide a unified voice as part of the Galaxy governance. Indeed, recognizing the growing gap between users and developers within the expanding Galaxy community, the User Interface/User Experience (UI/UX) team has established a strong connection with these SIGs, interviewing users, establishing platform user experience benchmarking and testing interventions. Insights gathered from training events—often, if not always run in part by SIGs—are then channelled back to the UI/UX team through interviews and presentations at meetings, fostering a continuous improvement cycle.

A few recent examples of SIG outputs include:

- ***Vertebrate Genome Project***: Galaxy has demonstrated its utility in support of the VGP through the publication of the version 2.1 VGP assembly workflow (22). Using data from VGP and ERGA, the workflow has generated 51 genomes, from 4 amphibian, 15 bird, 10 fish, 14 mammal and 8 reptile species.
- ***Computational proteomics***: In close collaboration with AnalystSuite, interactive tools, such as LFQanalyst, for the visualisation and exploration of data are available on Galaxy (23).
- ***Human genetics***: Galaxy has increased support for human genetics, especially with new workflows for discovering and interpreting genetic variations for use within the NHGRI AnVIL (24) and NCI Firecloud (25) environments.

### Environmental impact

**Figure 5.**
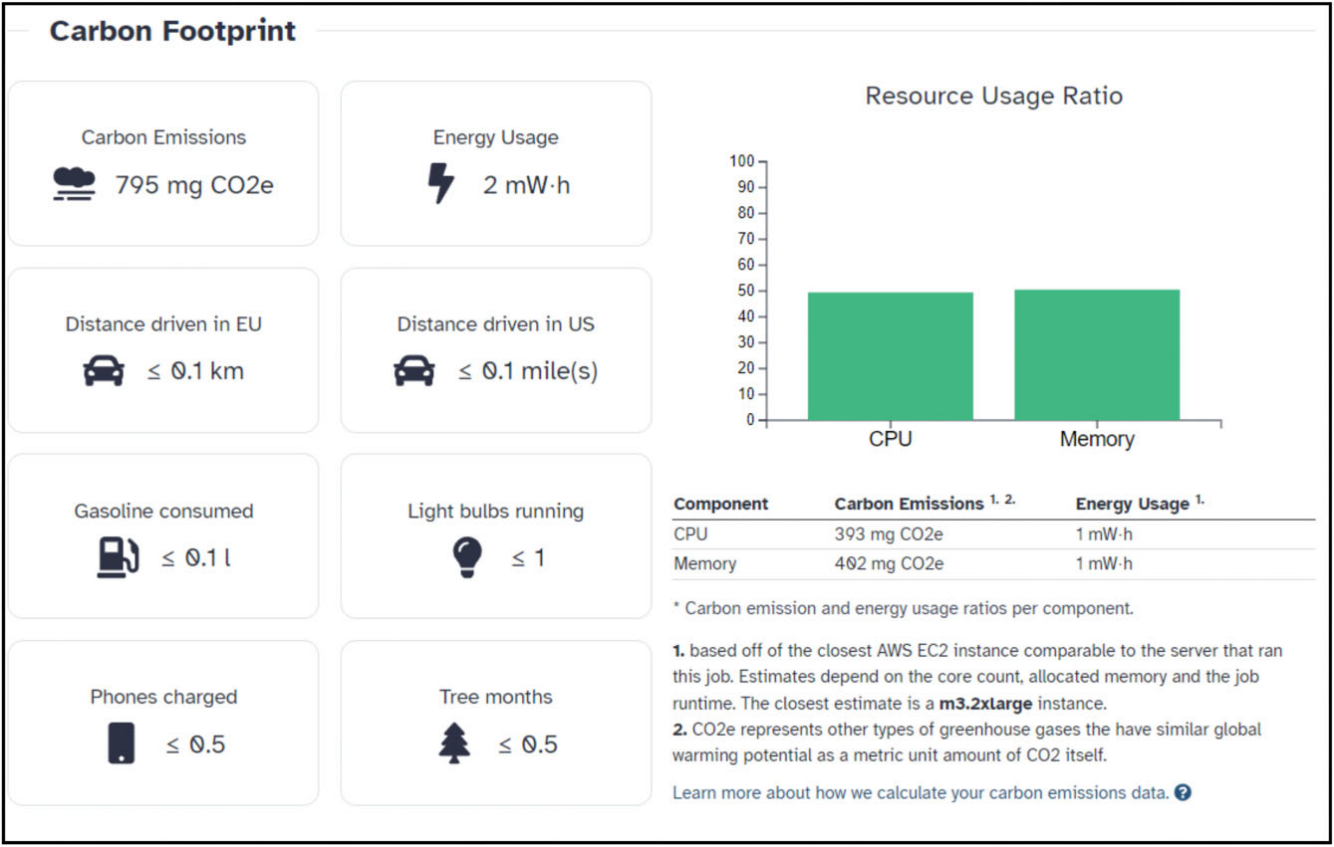
Example of estimated CO_2_ production associated with a Galaxy job. Sourced from a MSstats job on Galaxy Australia (https://usegalaxy.org.au), running Galaxy code release 23.1.

As a responsible service provider to a resource intensive global community, the Galaxy Project helps clarify the environmental impact of conducting research, by showing the estimated production of CO_2_ for every job executed (Figure 5).

## Future plans

Galaxy continues to advance on new software technologies, new scientific datatypes and applications, and new paradigms for collaborative research. This includes; shared Pulsar servers, utilization of scratch storage and broadening research domain support. Ultimately this is driven by the core values to support accessible, reproducible, and transparent user-driven research. This includes ensuring that Galaxy can integrate with external data sources, computational infrastructure and third party analysis tools, and building a strong, welcoming, collaborative community of users, developers, and stakeholders who contribute to the ongoing improvement of the Galaxy platform and ecosystem. The Galaxy Project relies extensively on our vibrant user community, and we continuously monitor, adapt and evolve to meet the changing needs of the open science research community.

## Data Availability

Galaxy is freely available at https://galaxyproject.org.

## Appendix

Corresponding author: Gareth **Price**(g.price@uq.edu.au)

Co-corresponding authors: Anton **Nekrutenko** (anton@nekrut.org), Björn A. **Grüning** (bjoern.gruening@gmail.com), Michael C. **Schatz** (mschatz@cs.jhu.edu)

Contributors (alphabetical)

Linelle Ann L Abueg^46^, Enis Afgan^31^, Olivier Allart^2^, Ahmed H Awan^31^, Wendi A Bacon^45^, Dannon Baker^31^, Madeline Bassetti^39^, Bérénice Batut^3^, Matthias Bernt^51^, Daniel Blankenberg^32^, Aureliano Bombarely^27^, Anthony Bretaudeau^26^, Catherine J Bromhead^49^, Melissa L Burke^50, 5^, Patrick K Capon^49, 5^, Martin Čech^38^, María Chavero-Díez^6^, John M Chilton^38^, Tyler J Collins^31^, Frederik Coppens^60^, Nate Coraor^38^, Gianmauro Cuccuru^25^, Fabio Cumbo^8^, John Davis^31^, Paul F De Geest^60^, Willem de Koning^17^, Martin Demko^33^, Assunta DeSanto^38^, José Manuel Domínguez Begines^3^, Maria A Doyle^55^, Bert Droesbeke^52, 60^, Anika Erxleben-Eggenhofer^3^, Melanie C Föll^3,^ Giulio Formenti^46^, Anne Fouilloux^42^, Rendani Gangazhe^3^, Tanguy Genthon^35^, Jeremy Goecks^34^, Alejandra N Gonzalez Beltran^41^, Nuwan A Goonasekera^49^, Nadia Goué^7^, Timothy J Griffin^58^, Björn A Grüning^3,^ Aysam Guerler^31^, Sveinung Gundersen^59^, Ove Johan Ragnar Gustafsson^49^, Christina Hall^49, 5^, Thomas W Harrop^49^, Helge Hecht^33^, Alireza Heidari^3^, Tillman Heisner^3^, Florian Heyl^21^, Saskia Hiltemann^3, 17^, Hans-Rudolf Hotz^19^, Cameron J Hyde^39^, Pratik D Jagtap^58^, Julia Jakiela^48^, James E Johnson^58^, Jayadev Joshi^8,^ Marie Jossé^9^, Khaled Jum’ah^47^, Matúš Kalaš^54^, Katarzyna Kamieniecka^47^, Tunc Kayikcioglu^3^, Markus Konkol^1^, Leonid Kostrykin^24^, Natalie Kucher^31^, Anup Kumar^3^, Mira Kuntz^3^, Delphine Lariviere^38^, Ross Lazarus^20^, Yvan Le Bras^35^, Gildas Le Corguillé^44^, Justin Lee^30, 39^, Simone Leo^10^, Leandro Liborio^40, 41^, Romane Libouban^29^, David López Tabernero^3^, Lucille Lopez-Delisle^12^, Laila S Los^3^, Alexandru Mahmoud^23^, Igor Makunin^50^, Pierre Marin^18^, Subina Mehta^58^, Winnie Mok^49, 5^, Pablo A Moreno^4^, François Morier-Genoud^16^, Stephen Mosher^31^, Teresa Müller^3^, Engy Nasr^3^, Anton Nekrutenko^38^, Tiffanie M Nelson^22, 5^, Asime J Oba^56^, Alexander Ostrovsky^31^, Polina V Polunina^3^, Krzysztof Poterlowicz^47^, Elliott J Price^33^, Gareth R Price^50, 39^, Helena Rasche^17^, Bryan Raubenolt^32^, Coline Royaux^43^, Luke Sargent^37^, Michelle T Savage^31^, Volodymyr Savchenko^16^, Denys Savchenko^53^, Michael C Schatz^31^, Pauline Seguineau^35^, Beatriz Serrano-Solano^14^, Nicola Soranzo^11^, Sanjay Kumar Srikakulam^3^, Keith Suderman^31^, Anna E Syme^49^, Marco Antonio Tangaro^28^, Jonathan A Tedds^13^, Mehmet Tekman^3^, Wai Cheng (Mike) Thang^50, 39^, Anil S Thanki^15^, Michael Uhl^3^, Marius van den Beek^38^, Deepti Varshney^3^, Jenn Vessio^31^, Pavankumar Videm^3^, Greg Von Kuster^38^, Gregory R Watson^36^, Natalie Whitaker-Allen^31^, Uwe Winter^49, 5^, Martin Wolstencroft^47^, Federico Zambelli^57^, Paul Zierep^3^, Rand Zoabi^3^

Affiliations

1. 52°North Spatial Information Research, North Rhine-Westphalia 48147, Germany

2. AARNet, Queensland 4104, Australia

3. Albert-Ludwigs-Universität Freiburg, Baden Württemberg 79110, Germany

4. AstraZeneca, Cambridgeshire CB2 0AA, UK

5. Australian Biocommons, Victoria 3052, Australia

6. Barcelona Supercomputing Center, Catalonia 08902, Spain

7. Clermont Auvergne University, Auvergne-Rhône-Alpes 63000, France

8. Cleveland Clinic, Ohio 44106, USA

9. CNRS - Data Terra, Brittany 29200, France

10. CRS4, Cagliari 09050, Italy

11. Earlham Institute, East of England and East Anglia NR4 7UZ, UK

12. Ecole Polytechnique Fédérale de Lausanne (EPFL), Vaud 1015, Switzerland

13. ELIXIR, Cambridgeshire CB10 1SD, UK

14. EMBL, Baden-Württemberg 69117, Germany

15. EMBL’s European Bioinformatics Institute (EMBL-EBI), Cambridgeshire CB10 1SD, UK

16. EPFL, Vaud 1004, Switzerland

17. Erasmus Medical Center, South Holland 3015 GD, The Netherlands

18. French Institute of Bioinformatics, Auvergne-Rhône-Alpes 63170, France

19. Friedrich Miescher Institute for Biomedical Research, Basel-Stadt 4058, Switzerland

20. Galaxy Project, New South Wales 2026, Australia

21. German Cancer Research Center, Baden-Württemberg 69120, Germany

22. Griffith University, New South Wales 2299, Australia

23. Harvard Medical School, Massachusetts 01082, USA

24. Heidelberg University, Baden-Württemberg 69120, Germany

25. HumanTechnopole, Lombardy 20157, Italy

26. IGEPP, INRAE, Institut Agro, Univ Rennes, Brittany 35042, France

27. Institute for Plant Cellular and Molecular Biology (IBMCP), Valencia 46001, Spain

28. Institute of Biomembranes, Bioenergetics and Molecular Biotechnologies, National Research Council (CNR), Apulia 70126, Italy

29. IRISA, Brittany 35042, France

30. James Cook University, Queensland 4814, Australia

31. Johns Hopkins University, Maryland 21218, USA

32. Lerner Research Institute, Cleveland Clinic, Ohio 44106, USA

33. Masaryk University, South Moravian 60200, Czech Republic

34. Moffitt Cancer Center, Florida 33612, USA

35. Museum national d’Histoire naturelle, Brittany 29900, France

36. Oak Ridge National Laboratory, Tennessee 37380, USA

37. Oregon Health & Science University, Oregon 97239, USA

38. Pennsylvania State University, Pennsylvania 16802, USA

39. Queensland Cyber Infrastructure Foundation, Queensland 4072, Australia

40. Rutherford Appleton Laboratory, UKRI, Oxfordshire OX11 0QX, UK

41. Science and Technology Facilities Council, Oxfordshire OX11 0QX, UK

42. Simula Research Laboratory, Oslo 0164, Norway

43. Sorbonne Université, Brittany 29900, France

44. Station Biologique de Roscoff - Sorbonne Université/CNRS, Brittany 29680, France

45. The Open University, Buckinghamshire MK7 6AA, UK

46. The Rockefeller University, New York 10021, USA

47. The University of Bradford, West Yorkshire BD7 1DP, UK

48. The University of Edinburgh, Edinburgh EH9 3FJ, UK

49. The University of Melbourne, Victoria 3052, Australia

50. The University of Queensland, Queensland 4072, Australia

51. UFZ Leipzig, Saxony 04318, Germany

52. UGent, East Flanders 9000, Belgium

53. Université Paris Cité, Île-de-France 75013, France

54. University of Bergen, Vestland 5008, Norway

55. University of Limerick, Munster V94 T9PX, Ireland

56. University of Maiduguri, Borno State 600004, Nigeria

57. University of Milan, Lombardy 20133, Italy

58. University of Minnesota, MN 55455, USA

59. University of Oslo, Oslo 0316, Norway

60. Vlaams Instituut voor Biotechnologie, East Flanders 9000, Belgium

## References

[1] Giardine B., Riemer C., Hardison R.C., Burhans R., Elnitski L., Shah P., Zhang Y., Blankenberg D., Albert I., Taylor J. et al. Galaxy: a platform for interactive large-scale genome analysis. Genome Res. 2005; 15:1451–1455. 16169926 10.1101/gr.4086505 PMC1240089

[2] Galaxy Community The Galaxy platform for accessible, reproducible and collaborative biomedical analyses: 2022 update. Nucleic Acids Res. 2022; 50:W345–W351. 35446428 10.1093/nar/gkac247 PMC9252830

[3] Black M., Lamothe L., Eldakroury H., Kierkegaard M., Priya A., Machinda A., Khanduja U.S., Patoliya D., Rathi R., Nico T.P.C. et al. EDAM: the bioscientific data analysis ontology (update 2021). F1000Research. 2022; 10.7490/f1000research.1118900.1.

[4] Rhie A., McCarthy S.A., Fedrigo O., Damas J., Formenti G., Koren S., Uliano-Silva M., Chow W., Fungtammasan A., Kim J. et al. Towards complete and error-free genome assemblies of all vertebrate species. Nature. 2021; 592:737–746. 33911273 10.1038/s41586-021-03451-0 PMC8081667

[5] Lewin H.A., Robinson G.E., Kress W.J., Baker W.J., Coddington J., Crandall K.A., Durbin R., Edwards S.V., Forest F., Gilbert M.T.P. et al. Earth BioGenome Project: sequencing life for the future of life. Proc. Natl. Acad. Sci. U.S.A. 2018; 115:4325–4333. 29686065 10.1073/pnas.1720115115 PMC5924910

[6] Marx-Stoelting P., Rivière G., Luijten M., Aiello-Holden K., Bandow N., Baken K., Cañas A., Castano A., Denys S., Fillol C. et al. A walk in the PARC: developing and implementing 21st century chemical risk assessment in Europe. Arch. Toxicol. 2023; 97:893–908. 36645448 10.1007/s00204-022-03435-7 PMC9968685

[7] Jumper J., Evans R., Pritzel A., Green T., Figurnov M., Ronneberger O., Tunyasuvunakool K., Bates R., Žídek A., Potapenko A. et al. Highly accurate protein structure prediction with AlphaFold. Nature. 2021; 596:583–589. 34265844 10.1038/s41586-021-03819-2 PMC8371605

[8] OpenAI A.J., Adler S., Agarwal S., Ahmad L., Akkaya I., Aleman F.L., Almeida D., Altenschmidt J., Altman S. et al. GPT-4 technical report. 2023; arXiv doi:15 March 2023, preprint: not peer reviewed https://arxiv.org/abs/2303.08774.

[9] Mirdita M., Schütze K., Moriwaki Y., Heo L., Ovchinnikov S., Steinegger M. ColabFold: making protein folding accessible to all. Nat. Methods. 2022; 19:679–682. 35637307 10.1038/s41592-022-01488-1 PMC9184281

[10] de Koning W., Miladi M., Hiltemann S., Heikema A., Hays J.P., Flemming S., van den Beek M., Mustafa D.A., Backofen R., Grüning B. et al. NanoGalaxy: nanopore long-read sequencing data analysis in Galaxy. Gigascience. 2020; 9:giaa105. 33068114 10.1093/gigascience/giaa105 PMC7568507

[11] Cox J., Mann M. MaxQuant enables high peptide identification rates, individualized p.p.b.-range mass accuracies and proteome-wide protein quantification. Nat. Biotechnol. 2008; 26:1367–1372. 19029910 10.1038/nbt.1511

[12] Zheng G.X.Y., Terry J.M., Belgrader P., Ryvkin P., Bent Z.W., Wilson R., Ziraldo S.B., Wheeler T.D., McDermott G.P., Zhu J. et al. Massively parallel digital transcriptional profiling of single cells. Nat. Commun. 2017; 8:14049. 28091601 10.1038/ncomms14049 PMC5241818

[13] Solovyev V., Kosarev P., Seledsov I., Vorobyev D. Automatic annotation of eukaryotic genes, pseudogenes and promoters. Genome Biol. 2006; 7:S10. 10.1186/gb-2006-7-s1-s10 PMC1810547 16925832

[14] Grüning B., Dale R., Sjödin A., Chapman B.A., Rowe J., Tomkins-Tinch C.H., Valieris R., Köster J. Bioconda Team Bioconda: sustainable and comprehensive software distribution for the life sciences. Nat. Methods. 2018; 15:475–476. 29967506 10.1038/s41592-018-0046-7 PMC11070151

[15] Wilkinson M.D., Dumontier M., Aalbersberg I.J.J., Appleton G., Axton M., Baak A., Blomberg N., Boiten J.-W., da Silva Santos L.B., Bourne P.E. et al. The FAIR Guiding Principles for scientific data management and stewardship. Sci. Data. 2016; 3:160018. 26978244 10.1038/sdata.2016.18 PMC4792175

[16] Yuen D., Cabansay L., Duncan A., Luu G., Hogue G., Overbeck C., Perez N., Shands W., Steinberg D., Reid C. et al. The Dockstore: enhancing a community platform for sharing reproducible and accessible computational protocols. Nucleic Acids Res. 2021; 49:W624–W632. 33978761 10.1093/nar/gkab346 PMC8218198

[17] Rehm H.L., Page A.J.H., Smith L., Adams J.B., Alterovitz G., Babb L.J., Barkley M.P., Baudis M., Beauvais M.J.S., Beck T. et al. GA4GH: international policies and standards for data sharing across genomic research and healthcare. Cell Genom. 2021; 1:100029. 35072136 10.1016/j.xgen.2021.100029 PMC8774288

[18] Hiltemann S., Rasche H., Gladman S., Hotz H.-R., Larivière D., Blankenberg D., Jagtap P.D., Wollmann T., Bretaudeau A., Goué N. et al. Galaxy Training: a powerful framework for teaching!. PLoS Comput. Biol. 2023; 19:e1010752. 36622853 10.1371/journal.pcbi.1010752 PMC9829167

[19] Mölder F., Jablonski K.P., Letcher B., Hall M.B., Tomkins-Tinch C.H., Sochat V., Forster J., Lee S., Twardziok S.O., Kanitz A. et al. Sustainable data analysis with Snakemake. F1000Res. 2021; 10:33. 34035898 10.12688/f1000research.29032.1 PMC8114187

[20] Rasche H., Hyde C., Davis J., Gladman S., Coraor N., Bretaudeau A., Cuccuru G., Bacon W., Serrano-Solano B., Hillman-Jackson J. et al. Training infrastructure as a service. Gigascience. 2022; 12:giad048. 37395629 10.1093/gigascience/giad048 PMC10316688

[21] Soiland-Reyes S., Sefton P., Crosas M., Castro L.J., Coppens F., Fernández J.M., Garijo D., Grüning B., La Rosa M., Leo S. et al. Packaging research artefacts with RO-Crate. Data Sci. 2022; 5:97–138.

[22] Larivière D., Abueg L., Brajuka N., Gallardo-Alba C., Grüning B., Ko B.J., Ostrovsky A., Palmada-Flores M., Pickett B.D., Rabbani K. et al. Scalable, accessible and reproducible reference genome assembly and evaluation in Galaxy. Nat. Biotechnol. 2024; 42:367–370. 38278971 10.1038/s41587-023-02100-3 PMC11462542

[23] Mehta S., Bernt M., Chambers M., Fahrner M., Föll M.C., Gruening B., Horro C., Johnson J.E., Loux V., Rajczewski A.T. et al. A galaxy of informatics resources for MS-based proteomics. Expert Rev. Proteomics. 2023; 20:251–266. 37787106 10.1080/14789450.2023.2265062

[24] Schatz M.C., Philippakis A.A., Afgan E., Banks E., Carey V.J., Carroll R.J., Culotti A., Ellrott K., Goecks J., Grossman R.L. et al. Inverting the model of genomics data sharing with the NHGRI Genomic Data Science Analysis, Visualization, and Informatics Lab-space. Cell Genom. 2022; 2:100085. 35199087 10.1016/j.xgen.2021.100085 PMC8863334

[25] Birger C., Hanna M., Salinas E., Neff J., Saksena G., Livitz D., Rosebrock D., Stewart C., Leshchiner I., Baumann A. et al. FireCloud, a scalable cloud-based platform for collaborative genome analysis: strategies for reducing and controlling costs. 2017; bioRxiv doi:03 Novemberv 2017, preprint: not peer reviewed 10.1101/209494.

[26] Nekrutenko A., Schatz M.C. In memory of James Taylor: the birth of Galaxy. Genome Biol. 2020; 21:105. 32354350 10.1186/s13059-020-02016-0 PMC7193333

